# Surgery for fibroadenoma arising from axillary accessory breast

**DOI:** 10.1186/s12905-021-01278-5

**Published:** 2021-04-07

**Authors:** Sung Ryul Lee

**Affiliations:** Department of Surgery, Damsoyu Hospital, 234 Hakdong-ro, Gangnam-gu, Seoul, Republic of Korea

**Keywords:** Axillary accessory breast, Fibroadenoma, Accessory mammary gland

## Abstract

**Background:**

Patients with fibroadenomas in axillary accessory breasts (AABs) have a palpable mass, cyclic axillary pain, and aesthetic concerns that must be addressed. We compared the baseline patient characteristics, AAB characteristics, and surgical outcomes of patients with AABs with and without fibroadenomas undergoing surgical excision. We also monitored the patients for recurrence of axillary fibroadenomas.

**Methods:**

This retrospective study involved 2310 women who underwent AAB excision from 2014 to 2019. Patients with and without a palpable fibroadenoma were divided into a fibroadenoma group and non-fibroadenoma group, respectively. All patients underwent complete excision of accessory mammary gland (AMG) tissue, including fibroadenomas in the AABs. We removed the fibroadenoma and the AMG tissue with a minimal axillary incision.

**Results:**

Thirty-nine patients had a palpable fibroadenoma in the AAB, and all patients in the fibroadenoma group had cyclic axillary pain and a palpable axillary mass. There were no significant differences in the patients’ age, weight of the AMG tissue, liposuction volume, or fibroadenoma laterality between the two groups. The body mass index in the fibroadenoma group was lower than that in the non-fibroadenoma group (19.9 vs. 22.3 kg/m^2^, respectively; *P* < 0.000). Concurrent fibroadenoma excision in a normal breast on the chest was performed more often in the fibroadenoma group than in the non-fibroadenoma group (35.9% (14/39) vs. 4.1% (92/2271), respectively; *P* < 0.000). The mean fibroadenoma size was 2.1 cm (range, 1.1–9.1 cm). All patients were satisfied with the degree of postoperative pain relief, disappearance of palpable lesions, and cosmetic improvement. No patients developed fibroadenoma recurrence.

**Conclusions:**

Complete excision of the AMG tissue and fibroadenoma is appropriate in patients with an AAB with a fibroadenoma. Surgeons should also consider the high incidence of concurrent fibroadenomas in the normal breasts on the chest.

## Background

An axillary accessory breast (AAB) occurs in 2–6% of women [1], and some patients require treatment for associated cyclic axillary pain or aesthetic concerns [2, 3]. Rarely, patients present with a chief complaint of a palpable nonpainful axillary mass, which has been described in case reports as a fibroadenoma [4]. Periodic enlargement of an AAB and cyclic pain are the primary reasons for surgical treatment. A palpable mass may also be an indication for surgical treatment. Lee et al. established an AAB classification system based on the severity of the external appearance of the AAB and recommended treatment by complete accessory mammary gland (AMG) excision with liposuction of the supramammary fat layer [5, 6]. To the best of our knowledge, although several case reports describing treatment for fibroadenomas arising from AABs have been published, no guidelines have been published and no large studies have been performed. Fibroadenomas in a normal breast on the chest (chest normal breast (CNB)) are usually asymptomatic; however, patients with fibroadenomas in an AAB may have cyclic pain and aesthetic concerns as typical symptoms of an AAB secondary to the presence of AMG tissue. For successful treatment of fibroadenomas in an AAB, we must determine whether the problem is limited to the fibroadenoma or is accompanied by pain and aesthetic concerns caused by the AMG tissue. Treatment of a palpable fibroadenoma in the CNB involves excision of the fibroadenoma and preservation of the mammary gland [7], but treatment of a fibroadenoma in an AAB should resolve the pain and aesthetic concerns caused by the AMG tissue. Therefore, complete excision of both the AMG tissue and fibroadenoma is necessary to treat fibroadenomas arising from an AAB. With this approach, it is possible to prevent fibroadenoma recurrence in the AAB.

In this study, we compared the baseline patient characteristics, AAB characteristics, and surgical outcomes of patients with AABs with and without fibroadenomas undergoing surgical excision. We also monitored the patients for recurrence of axillary fibroadenomas.

## Methods

**Fig. 1:**
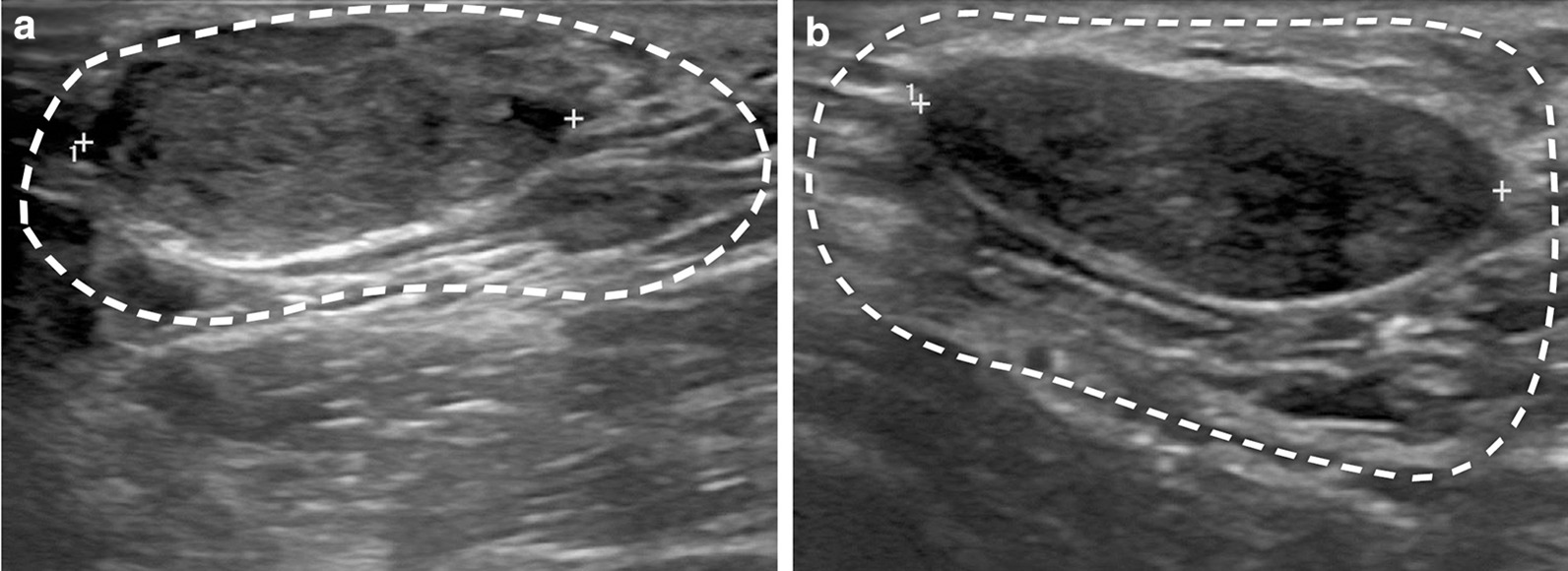
Sonographic findings of a fibroadenoma in an axillary accessory breast. **a** Accessory mammary gland (AMG) (dashed outline) surrounding an oval-shaped fibroadenoma. In the image, the top of the fibroadenoma is close to the skin, and at the bottom of the fibroadenoma, the AMG is visible surrounding the fibroadenoma. **b** AMG (dashed outline) surrounding an elliptical-shaped fibroadenoma. The AMG surrounds the fibroadenoma

We retrospectively analyzed the data of 2310 patients with an AAB treated in Damsoyu Hospital, Seoul, Republic of Korea from January 2014 to October 2019. The inclusion criteria for patients with an AAB who were candidates for surgical treatment were a > 3-year history of either or both of the following: (1) AAB enlargement and axillary pain and/or (2) persistent AAB enlargement and related psychosocial/emotional distress and (3) no malignant tumor in the CNB or AAB. Classification of the AAB was performed in accordance with the Damsoyu–Lee classification [5]. All patients underwent ultrasonography and pathological confirmation of lesions in the CNB and AAB. The ultrasonographic findings of a fibroadenoma in the AAB were characterized by the same oval hypoechoic appearance as seen with a fibroadenoma in the CNB (Fig. 1). The axillary tail of Spence connected to the CNB was excluded from the AAB. All patients with a Breast Imaging Reporting and Data System (BIRADS) category ≥ 4 mass in the CNB or AAB underwent core biopsy before surgery. After confirming the pathology by preoperative core biopsy, all ≥ 2-cm palpable fibroadenomas in the CNB were removed during AAB excision. Lesions of BIRADS category < 3 were followed up regularly. The fibroadenoma diagnoses were confirmed in the post-excision final pathologic examination. When cancer was histopathologically diagnosed, we referred the patient to a tertiary center. In this study, 14 patients had cancer in the CNB and no patients had cancer in the AAB. We divided the patients into a fibroadenoma group (n = 39) and a non-fibroadenoma group (n = 2271) according to the presence of a fibroadenoma in the AAB. All patients underwent complete excision of the AMG tissue, including the fibroadenoma.

We collected the following data from the patients’ medical records: age at surgery; body mass index; symptom characteristics, including onset; type and duration of surgery; histopathological results; size, location, and number of fibroadenomas; AMG tissue weight; liposuction volume; postoperative complications; and satisfaction score. The onset of AAB symptoms was divided into two categories: after puberty (from the time of puberty, when secondary sexual characteristics appeared and menstruation began) and after pregnancy (no AAB symptoms before marriage; AAB symptoms appeared only after pregnancy). All patients completed post-hoc satisfaction surveys evaluating their appearance and axillary pain 6 months after surgery. We evaluated fibroadenoma recurrence at the AAB site and overall satisfaction with the decreased level of axillary pain and alleviation of aesthetic concerns using a 5-point Likert scale in all patients [8].

### Surgical technique and follow-up

**Fig. 2:**
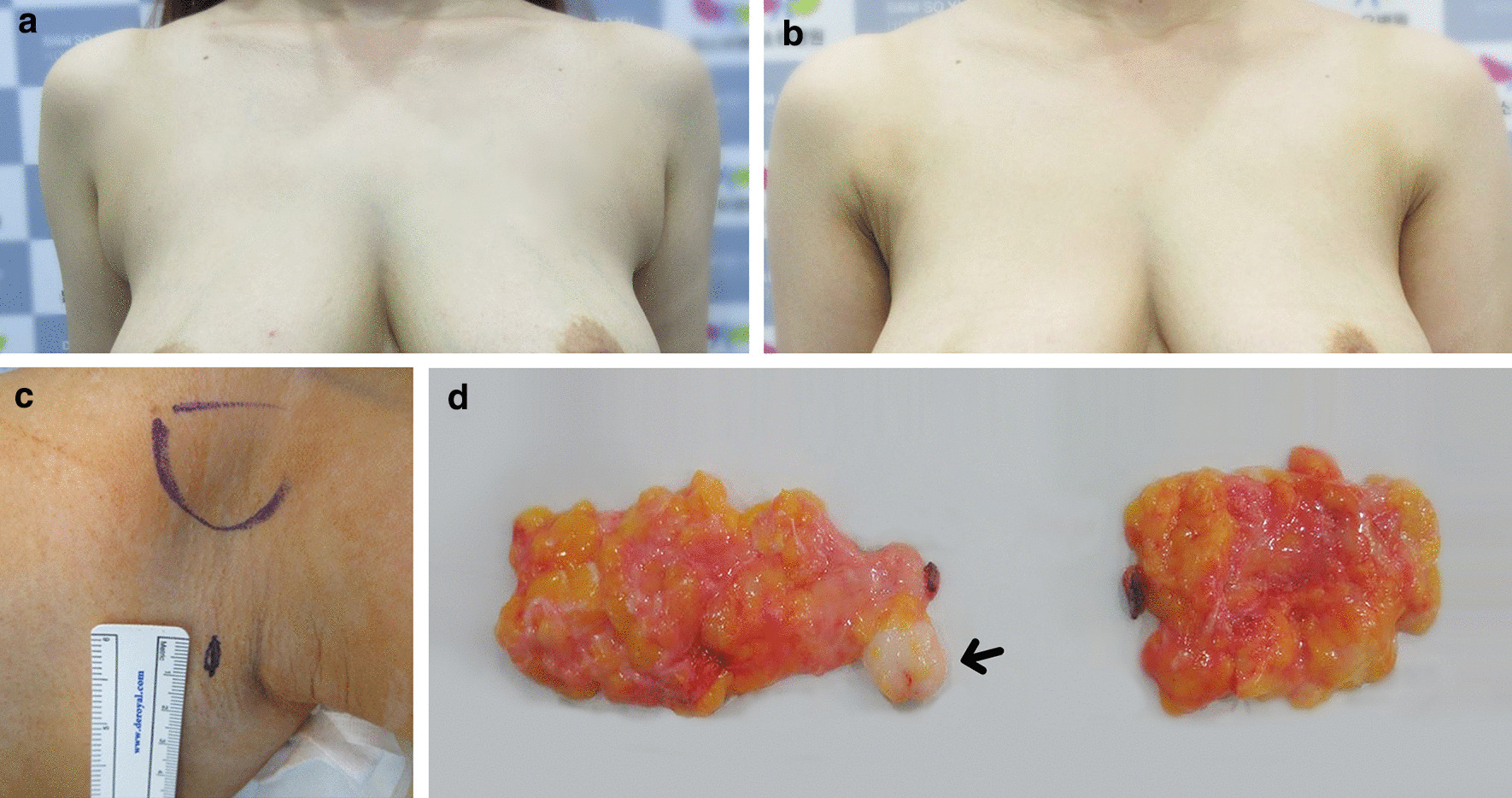
Fibroadenoma measuring 2.5 cm in an axillary accessory breast (AAB) in a 38-year-old woman who underwent surgery. **a** Preoperative frontal appearance with arms adducted. **b** Postoperative frontal view 2 weeks postoperatively. **c** The 1-cm incision along the axillary skin crease. **d** Accessory mammary gland tissue was removed through the 1-cm incision. The image on the left shows the fibroadenoma (black arrow) and accessory mammary gland tissue removed from the right AAB, and the image on the right shows the accessory mammary gland tissue removed from the left AAB

**Fig. 3:**
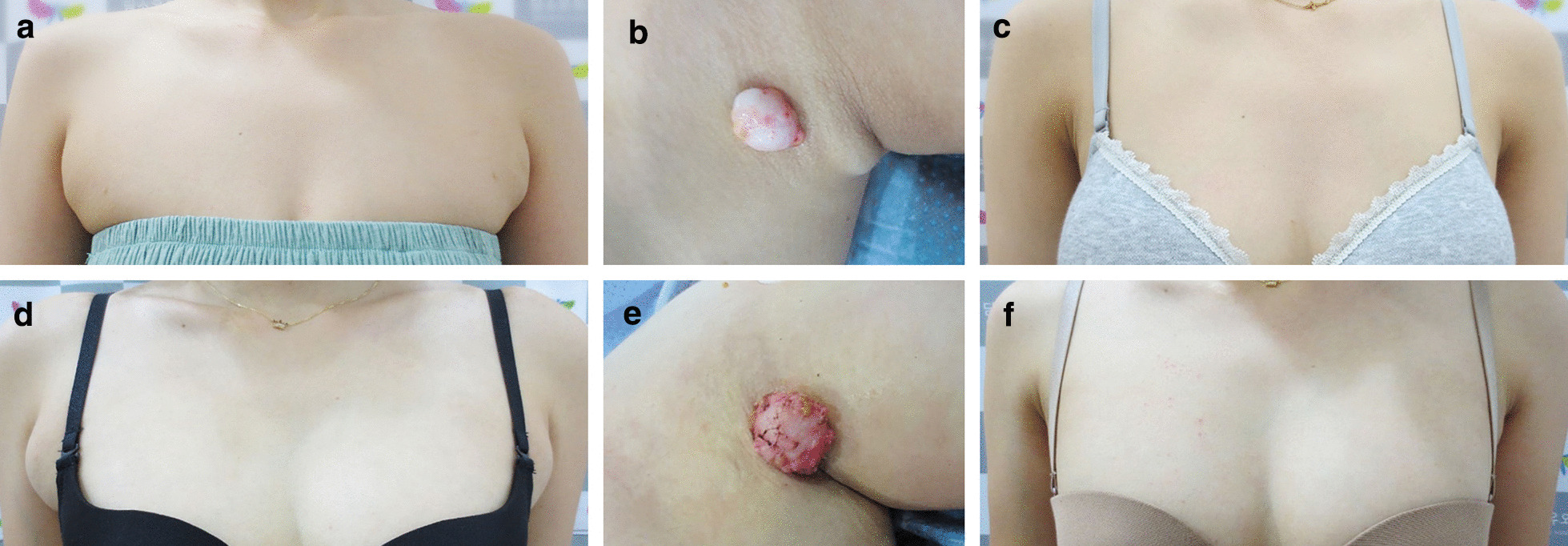
Photographs of two patients with fibroadenomas measuring more than 3 cm. **a** Preoperative axillary appearance of the first patient with her arms adducted. **b** A 3.2-cm fibroadenoma. **c** Frontal view of the patient 6 months postoperatively. **d** Preoperative right axillary appearance in the second patient with her arms adducted. **e** A 3.5-cm fibroadenoma. **f** Frontal view 6 months postoperatively

**Fig. 4:**
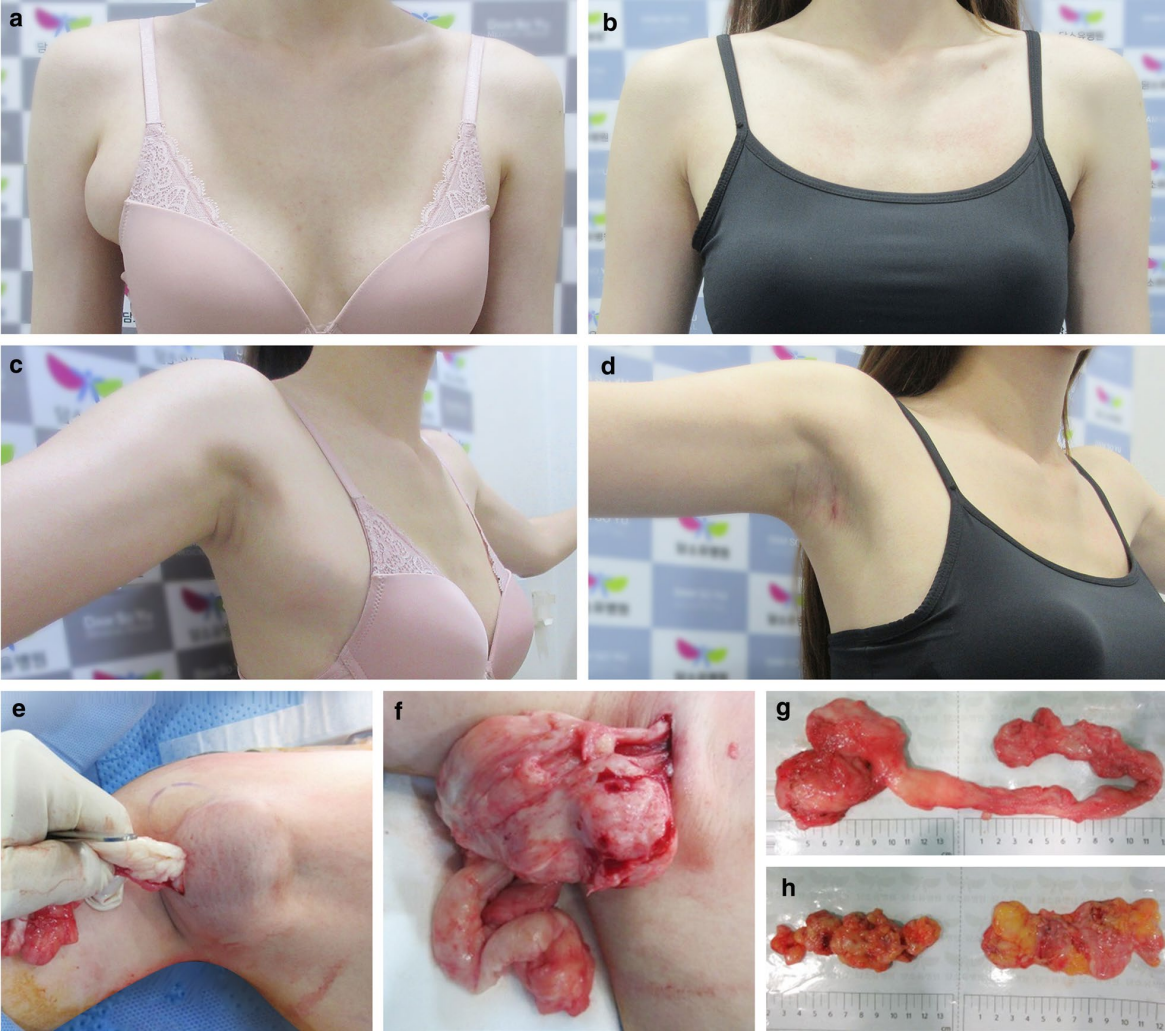
Photographs of a 28-year-old woman who underwent surgery to remove a 9.1-cm giant fibroadenoma arising from an axillary accessory breast (AAB). **a** Preoperative frontal appearance. The right side was misshapen by the giant fibroadenoma, and accessory mammary gland (AMG) tissue in both axillae was confirmed using ultrasonography. **b** Frontal view 6 months postoperatively. **c** Preoperative right axillary appearance with arms abducted. **d** Right axillary appearance 6 months postoperatively. **e** We removed the fibroadenoma by incising the skin and peeling away the fibroadenoma. **f** The fibroadenoma was completely removed from the axilla through a 1-cm skin incision. **g** Fibroadenoma specimen. **h** The AMG tissue was also removed through the 1-cm incision. The image on the left shows the AMG removed from the right AAB, and the image on the right shows the AMG removed from the left AAB

We adopted a previously reported AAB excision method through a 1-cm incision on the axillary crease and performed all procedures with the patients under general anesthesia [5, 6]. For small fibroadenomas, we performed en bloc resection with the AMG tissue (Fig. 2). For fibroadenomas measuring > 3 cm, we first excised the fibroadenoma and then excised the AMG tissue (Fig. 3) to reduce the severity of the incision scar. In patients with giant fibroadenomas, we removed the fibroadenoma by peeling to reduce the size of the resulting scar (Fig. 4). We first instilled a tumescent solution through the incision into the accessory breast tissue. This was followed by liposuction with a power-assisted device. After liposuction of the supramammary fat layer, we performed complete AMG tissue excision and closed the incision with subcuticular absorbable sutures. The need for redundant skin excision was determined after 6 months [6]. An external drain was not inserted. After closing the wound, we applied adhesive skin closures (Steri-Strip; 3 M Health Care, Maplewood, MN, USA) to the incision. Follow-up examinations were routinely performed at 1, 3, and 6 months postoperatively.

### Statistical analysis and assessment methods

All statistical analyses were performed using R software version 3.6.1 (R Development Core Team, Vienna; http://www.R-project.org). Continuous variables are presented as mean and range, and categorical variables are presented as frequency and percentage. The Shapiro–Wilk test was used to assess the normality of continuous variables. The t-test or Wilcoxon’s rank-sum test was used for continuous variables, and the χ^2^ test was used for categorical variables. A *P* value of ≤ 0.05 in the univariate analysis was considered statistically significant. We administered 6-month postoperative surveys evaluating the patients’ satisfaction with their appearance and axillary pain compared with the preoperative levels and measured the patients’ satisfaction with the scar, pain, and appearance separately using a 5-point Likert scale (1: continuous pain and very unsatisfactory appearance; 2: some pain and unsatisfactory appearance; 3: neutral; 4: little pain and satisfactory appearance; and 5: no pain and very satisfactory appearance) [8].

## Results

### Clinical characteristics and skin excision outcomes between fibroadenoma and non-fibroadenoma groups

The patients’ clinical characteristics and surgical outcomes are summarized in Table 1. We compared the baseline patient characteristics and AAB characteristics between the fibroadenoma and non-fibroadenoma groups and found no differences in age, AMG tissue weight, liposuction volume, family history, or fibroadenoma laterality between the two groups. The body mass index in the fibroadenoma group was lower than that in the non-fibroadenoma group (19.9 vs. 22.3 kg/m^2^, respectively; *P* < 0.000). All patients in the fibroadenoma group had axillary pain secondary to the AMG tissue, but there was no significant difference in pain between the two groups. The onset of AAB symptoms was more frequent after puberty in the fibroadenoma group (79.5%) than in the non-fibroadenoma group (57.0%) (*P* = 0.008), and the number of patients who underwent fibroadenoma excision in the CNB was greater in the fibroadenoma group than in the non-fibroadenoma group (35.9% (14/39) vs. 4.1% (92/2271), respectively; *P* < 0.000). Five patients had fibrocystic change in the AAB; none had a malignant tumor. The postoperative complication rate, redo operation rate, and satisfaction score were not significantly different between the two groups, and the satisfaction scores for aesthetic concerns and axillary pain were > 4.7 in both groups. No patients developed fibroadenoma recurrence in the axilla during the average follow-up period of 41 months.

**Table 1:** Clinical characteristics and surgical outcomes between fibroadenoma and non-fibroadenoma groups

	AAB with fibroadenoma (n = 39)	AAB without fibroadenoma (n = 2271)	*P*-value*
Age, years	34.3 (22–48)	33.8 (13–65)	0.687
Category			0.372
≤ 20	0 (0.0)	78 (3.4)	
21–40	27 (69.2)	1694 (74.6)	
41–60	12 (30.8)	490 (21.6)	
> 60	0 (0.0)	9 (0.4)	
BMI, kg/m^2^	19.9 (15.5–24.1)	22.3 (17.2–33.7)	< 0.000
Family history of AAB			0.585
None	37 (94.8)	2043 (89.9)	
Mother	1 (2.6)	136 (6.0)	
Siblings	1 (2.6)	92 (4.1)	
Symptoms			0.148
Palpable mass	0 (0.0)	167 (7.3)	
Pain and palpable mass	39 (100.0)	2104 (92.7)	
Onset of symptoms			0.008
After puberty	31 (79.5)	1294 (57.0)	
After pregnancy	8 (20.5)	977 (43.0)	
DL classification			0.288
I	29 (74.4)	1408 (62.0)	
A	1	230	
B	28	1170	
II	8 (20.5)	693 (30.5)	
A	2	110	
B	6	583	
III	2 (5.1)	170 (7.5)	
A	1	70	
B	1	100	
Concurrent fibroadenoma excision in the chest normal breast	14 (35.9)	92 (4.1)	< 0.000
Operation time, minutes	39.4 (30–60)	39.0 (30–60)	0.905
Weight of the accessory mammary gland, g	57.4 (25–157)	59.9 (10–204)	0.641
Liposuction volume, mL	301.7 (60–600)	338.6 (60–1200)	0.759
Other tumor	0 (0.0)	5 (0.2)	1.000
Fibrocystic disease	0	5	
Malignancy	0	0	
Postoperative complications	1 (2.6)	54 (2.4)	0.986
Hematoma	0	4	
Seroma	1	20	
Surgical site infection	0	1	
Skin ischemia	0	6	
Axillary numbness	0	3	
Hypertrophic scar	0	4	
Hyperpigmentation	0	10	
Irregular skin contour	0	6	
Redo operation	1 (2.6)	60 (2.6)	0.919
Redundant skin excision	0	50	
Remnant mammary gland	0	0	
Liposuction for irregular contour	1	6	
Scar revision for hypertrophic scar	0	4	
Postoperative fibroadenoma growth in axilla	0 (0.0)	0 (0.0)	
Satisfaction score^a^			
Appearance	4.7 (4–5)	4.7 (4–5)	0.544
Axillary pain	4.8 (4–5)	4.7 (4–5)	0.664
Overall	9.4 (8–10)	9.4 (9–10)	0.687
Follow-up period	41.6 (6–75)	46.1 (6–75)	0.376

Data are presented as mean (range), n (%), or n

^*^Most *P*-values represent comparisons between categorical variables, which were tested using the *χ*^2^ test. Continuous variables were tested using the Wilcoxon rank-sum test and t-test

^a^Satisfaction score was measured according to scar, pain, and cosmesis level scores 6 months postoperatively using a 5-point Likert scale

AAB, axillary accessory breast; BMI, body mass index; DL classification, Damsoyu–Lee classification

### Clinical characteristics of AABs with fibroadenomas

The characteristics and surgical outcomes of patients with a fibroadenoma in an AAB are summarized in Table 2. Fibroadenomas were present in bilateral AABs in 1 patient, in a right-sided AAB in 21 patients, and in a left-sided AAB in 17 patients. One patient had two fibroadenomas, and all other patients had a single fibroadenoma. The largest fibroadenoma measured 9.1 cm in diameter, and the mean fibroadenoma diameter was 2.1 cm (range, 1.1–9.1 cm). All patients were satisfied with the degree of postoperative pain relief, disappearance of palpable lesions, and cosmetic improvement. No patients developed fibroadenoma recurrence during follow-up.

**Table 2:** Clinical characteristics and surgical outcomes in patients with fibroadenomas in an AAB

	39 patients
Location of the fibroadenoma	
Right	21 (53.8)
Left	17 (43.6)
Bilateral	1 (2.6)
Number of fibroadenomas in unilateral AABs	
One	38 (97.4)
Two	1 (2.6)
Size of the fibroadenoma	2.1 (1.1–9.1)
1 to ≤ 2 cm	24 (61.5)
2 to ≤ 3 cm	10 (25.6)
3 to ≤ 4 cm	4 (10.3)
4 to ≤ 5 cm	0 (0.0)
> 5 cm	1 (2.6)
Recurrence of fibroadenoma	0 (0.0)
Follow-up period, months	41.6 (6–75)

Data are presented as mean (range) or n (%)

AAB, axillary accessory breast

## Discussion

Benign and malignant tumors may occur via pathological mechanisms in an AAB, as they do in the CNB [9]. Fibroadenoma is a common benign tumor characterized by a nodule of fibrous tissue with epithelial elements [10]. Although no large studies of fibroadenomas in AABs have been performed to date, one report indicated that the prevalence of fibroadenomas was 2.0% (11/540) among patients with AABs who underwent surgery [5]. Other benign tumors and cancers other than fibroadenoma have been reported in AABs, but such lesions are very rare [5, 11].

The main symptom of a fibroadenoma in an AAB is a palpable mass, and some patients present for evaluation because of a fear of cancer. When we examine patients with fibroadenomas, there are usually no symptoms directly related to the fibroadenoma; however, most patients have cyclic pain caused by the AMG tissue. In the present study, all 39 patients with a fibroadenoma in the AAB had cyclic pain. Such pain often occurs with cyclic swelling secondary to hormonal changes during menstruation and pregnancy, and the severity may be so great that the patients request surgical treatment [5, 12]. Several reports have stated that the aim of AAB surgery is to improve the patient’s appearance or to relieve axillary pain [2, 13]. Complete treatment of an AAB involves removing all AMG tissue [14], which may be accompanied by liposuction to reduce scars. Complete excision of the AMG tissue should be performed to prevent recurrence [12, 15]. In this study, the postoperative satisfaction surveys administered 6 months postoperatively revealed scores of ≥ 4.7 for axillary pain and aesthetic improvement in both groups, indicating that both the axillary cyclic pain and aesthetic concerns were almost eliminated.

A fibroadenoma may recur secondary to remnant tumor tissue regrowth after mass excision, or a new fibroadenoma may develop in the remaining AMG tissue. One study revealed a 15% recurrence rate 5 years after fibroadenoma removal in the CNB [16]. In the current study, the mean follow-up period was 48.2 months, and no patients developed fibroadenoma recurrence. Eliminating all AMG tissue in addition to fibroadenoma excision can reduce fibroadenoma recurrence. Patients with a fibroadenoma in the CNB develop cancer more often than patients without a fibroadenoma [10], and patients with a fibroadenoma in the AAB may also develop cancer in the AAB. In our study, no patients had cancer in the AAB at the time of surgery, and no cancer developed in the axilla during the postoperative follow-up. Therefore, complete treatment of a fibroadenoma in an AAB requires both fibroadenoma excision and complete excision of the AMG tissue, which is the source of the fibroadenoma development and which causes axillary pain. This study is clinically meaningful in that it is the first large study to present a treatment guideline for fibroadenomas in AABs.

This study had several limitations. First, our hospital does not treat malignant tumors and does not study carcinoma because patients with breast cancer are referred to a tertiary center when breast cancer is diagnosed preoperatively. Additionally, our hospital does not provide overall treatment for breast disease; instead, it is an institution that specializes in AAB treatment. No statistics are available on the prevalence of AAB because our hospital does not perform breast examinations for other breast diseases. This is why the prevalence of AAB in the present study may seem misleadingly high. Ethnic and geographical differences should be investigated in future studies. Second, the number of patients with fibroadenomas was relatively small in this study. Thus, larger studies involving higher numbers of patients are necessary. Additionally, although accessory breasts may occur in areas other than the axilla, only AABs were included in this study. Finally, patients without cyclic pain may not seek medical care; therefore, the prevalence of fibroadenomas in AABs may be lower than indicated in our study.

## Conclusions

We recommend complete excision of the AMG tissue including the fibroadenoma, which is appropriate in patients with a fibroadenoma in an AAB. Patients with a fibroadenoma in an AAB are at increased risk of also having fibroadenomas in the CNB. Importantly, our results showed that patients with fibroadenoma(s) in an AAB experienced cyclic axillary pain and aesthetic concerns. Furthermore, eliminating all AMG tissue in addition to fibroadenoma excision can reduce the rate of fibroadenoma recurrence.

## Data Availability

The datasets used and/or analyzed during the current study are available from the corresponding author on reasonable request.

## Abbreviations

AAB: Axillary accessory breast
AMG: Accessory mammary gland
CNB: Chest normal breast
